# The University and Hospital Bulletin

**Published:** 1905-02

**Authors:** 


### The University and Hospital Bulletin

DEVOTED TO THE INTERESTS OF THE UNIVERSITY OF BUFFALO AND THE HOSPITALS.

EDITED BY
NELSON W. WILSON, M. D.

*Assistants:*
E. R. McGuire, M. D., Burton T. Simpson, M. D.

Under the auspices of the Faculty of the Medical Department of the University

M. D. Mann, A. M., M. D., Dean,
Charles G. Stockton, M. D.,
John Parmenter, M. D.,
Eli H. Long, M. D.,
Roswell Park, A. M., M. D., LL. D.,
Charles Cary, M. D.,
Herbert M. Hill, A. M., Ph. D.,
Herbert U. Williams, M. D.

AND

*Buffalo General Hospital*
Henry Reed Hopkins, M. D.

*Hospital of the Sisters of Charity*
Eugene A. Smith, M. D.

*German Hospital*
Herman E. Hayd, M. D.

### Department of Anatomy, University of Buffalo.

I have been requested to prepare an article dealing with the teaching of anatomy in the University of Buffalo. As a preface to this I would like to say that in the departments of physiology, histology and anatomy the work has been planned to correlate these subjects so far as is possible. A modified concentration of anatomy and physiology is also adopted; during the first semester the greater number of hours are devoted to anatomy; during the second semester physiology replaces the extra hours which were devoted to anatomy.

The general plan of teaching is largely practical. The student is encouraged in every way to study from his work on the cadaver and from seeing and handling actual specimens, the knowledge acquired through the sense of sight being much more lasting than that gained by any other means.

A systematic method of consideration being one of the essentials of successful study, the student is given the following outline to follow as being applicable to nearly all anatomical structures, and the demonstrators use the same in class work: (1) situation and form; (2) size, weight or extent; (3) component parts for nerve and blood supply; (5) relations; (6) function or action; (7) clinical significance and application.

That the instructor may come into closer personal relation with the student the class is divided into sections, and the same demonstration is given to each section separately, it being much more satisfactory to present a subject and specimens to a class of twenty-five than to one of fifty or seventy-five.

The work is so arranged that while the student is receiving demonstrations on any particular part of the body he is at the same time dissecting that part in the laboratory. While studying and receiving demonstrations on the bones each student is supplied with a specimen for class work; in the case of the cranial bones each two or three students are given a specimen so that this difficult work is simplified as much as possible. Each member of the class is required to point out in the work on osteology, the structures mentioned by the instructor and a part of each period is devoted to a quiz or review of the preceding work.

In the freshman year the viscera are taught from the cadaver and attention is devoted particularly to the structure and relations of the different organs, leaving for the sophomore year the study of the surface anatomy and application of this course to medicine and surgery. In the case of an important and complicated organ like the heart, a pig's heart is supplied to each student and he dissects it as the demonstrator proceeds.

In the freshman year the course in anatomy comprises, therefore, demonstrations on the osteology and soft parts of the head and neck; the upper extremity; the lower and a course on the anatomy of the viscera of the thorax and abdomen, together with twelve hours a week dissection arranged as far as possible to cover the work mentioned. The instructors are requested while teaching the foregoing to explain to the student how this knowledge is required in medicine and surgery and to endeavor by this means to arouse interest and assist the memory. The subject of osteology is covered entirely in the first year, also myology and angiology, with the dissection of two parts at least, and if possible three, the upper, the lower and the thorax.

In the sophomore year the anatomy of the brain and spinal cord is taken up by a course of one demonstration per week during the entire term, being illustrated by actual dissections and specimens with the aid also of charts and blackboard drawings; in this course the demonstrations are somewhat in the nature of conferences between the instructor and students and, by the use of the inductive method, as much as possible is drawn out from the student.

The cranial and spinal nerves are taken up by themselves with also a few lectures on the sympathetic system and its very intimate relation to the cerebro-spinal is prominently brought out, and the far-reaching effect of this correlation, as instanced by many symptoms referred to parts at a distance from the seat of the trouble, is explained upon anatomical grounds and rendered more intelligible by the use of diagrams and blackboard drawings by the instructor; these drawings the student is required to repeat in quizzes and test examinations. One combined lecture and demonstration each week is also given to the class, in sections, on the advanced anatomy of the viscera of the thorax and abdomen, which is illustrated by models, charts, and also by the stripped living model, on which the students are required to outline the surface markings and location of viscera by means of crayons; in this way the student is familiarised with the practical application of the work. Previous to the course in obstetrics a series of about six demonstrations is given on the anatomy of the female pelvis by actual dissection of the cadaver; eight demonstrations are also given on the male genitourinary anatomy, these being illustrated by dissections, models, and drawings.

A few demonstrations are given on the lymphatic system, and the more important joints are taken up in class and their action in dislocations and fractures explained and shown by means of a set of prepared ligaments. In both years written test examinations are held from time to time for the benefit of the student and instructor, the results are recorded and taken into consideration in making up the final standing of the student at the end of the year.

In the anatomical laboratory the freshman student is assigned to the dissection of the part upon which he is receiving demonstrations in class; section I. being assigned to the upper, section II. to the lower extremity; upon completion of their respective courses and parts they are changed about. This plan obviates the great drawback of dissecting one part and receiving demonstrations on another at the same time.

Each student is given a card on which the regions of the part he is dissecting are outlined and, as these are satisfactorily dissected and shown to the demonstrator of anatomy, they are checked by him and it is essential that the student present a completed card to obtain his final examination in practical anatomy. This plan has been adopted for the purpose of preventing men from obtaining an examination without doing the full amount of dissection, and has been found to work very satisfactorily. In addition to this, a record of the standing of each student, as judged by the quizzes, is kept in the office of the demonstrator. These quizzes are given by either the demonstrator, one of the assistants in anatomy, or one of the advanced students who are appointed for this work; a complete record of all work done in the dissecting room is thus had and no student is eligible for his final examination in dissection until his record is satisfactory. The final examination on each part is given either in December or in April, by the demonstrator of anatomy, and consists of twenty to thirty minutes oral examination on the dissected cadaver and skeleton.

The sophomore student in the dissecting room is assigned to the head and abdomen for the reason that his work in class is thus correlated. A list of all the structures in the order in which they are met with in the work on the cadaver has been prepared and this forms the blank previously referred to, a few notes on the application of their work and some general anatomical laws, such as Hilton's rule for the nerve supply of joints are incorporated in this syllabus.

The criticism has often been made by the clinical teachers that, although the average medical student spends much time in the study of anatomy, when he comes to the bedside or the operating room he is unable to answer simple questions, or reason out for himself the results of certain disturbances in definite structures. This may be due not so much to his ignorance as to the fact that if the method of teaching as given in nearly all works is followed, he will learn anatomy synthetically, whereas nearly all the questions involving a knowledge of anatomy arising at the bedside in the examination of a patient will be from an analytical point of view, to which, unless previously instructed, he is perfectly unaccustomed; as for example, if asked what nerve supplies the peronei group of muscles, and what is their action? he will very probably be able to give a correct answer; but if shown a certain deformity of the foot and asked how this is accounted for, he may very possibly be unable to tell what muscles and nerve are involved unless his attention has been drawn to that aspect of the question when he is working on that part in the dissecting room. It is practically the same question but reversed.

Both in the dissecting room and in all classes, the teaching is directed to present the study of anatomy from an analytical as well as a synthetical point of view. Not only is this double presentation of the subject, so to speak, valuable from a practical standpoint but it also simplifies the memorising of a vast deal of work, by increasing the interest. From among those students who stand highest and who otherwise show an aptitude for the work, certain ones are chosen by the demonstrator of anatomy to act as prosectors to the assistants, their duties being to prepare dissections for class work, and also to quiz and assist the freshmen students in their work. These positions are considered an honor and the competition for them results in better work universally.

An additional spring course during the month of May lasting for five weeks, and consisting of work in dissection has been held for the past three years. During this term an opportunity is given those who care to take it, to do their dissection work when they can devote their entire attention to it, or to make up backwork, or repeat work on which they have failed to pass. The attendance on this course has averaged thirty.

In the junior year a course of two lectures a week is given to the whole class upon the applied anatomy of the whole body, but more especially those parts important in medicine and surgery, with illustrations from specimens, models, drawings and the living subject. JAMES A. GIBSON.

---

AT THE January meeting of the faculty of the medical department of the University of Buffalo, Dr. Montgomery Crockett was given a year's leave of absence.

---

DR. JAMES E. KING, instructor in obstetrics in the medical department of the University of Buffalo has been given a year's leave of absence by the faculty. Dr. King will spend the year in New York in study.

---

THE faculty appointed Dr. Burton T. Simpson to teach embryology and Dr. E. C. Mann, to teach obstetrics during Dr. King's absence.

---

AT THE staff meeting of the Buffalo General Hospital in January the resignation of Dr. James E. King, as assistant in the department of gynecology was accepted. The staff nominated to fill the vacancy Dr. E. C. Mann, the trustees later making the appointment.

